# Autologous cord blood cell therapy for neonatal hypoxic-ischaemic encephalopathy: a pilot study for feasibility and safety

**DOI:** 10.1038/s41598-020-61311-9

**Published:** 2020-03-12

**Authors:** Masahiro Tsuji, Mariko Sawada, Shinichi Watabe, Hiroyuki Sano, Masayo Kanai, Emi Tanaka, Satoshi Ohnishi, Yoshiaki Sato, Hisanori Sobajima, Takashi Hamazaki, Rintaro Mori, Akira Oka, Hiroyuki Ichiba, Masahiro Hayakawa, Satoshi Kusuda, Masanori Tamura, Makoto Nabetani, Haruo Shintaku

**Affiliations:** 1 Department of Food and Nutrition, Kyoto Women’s University, Kyoto, 605-8501 Japan; 2 Department of Regenerative Medicine and Tissue Engineering, National Cerebral and Cardiovascular Center, Suita, 565-8565 Japan; 3 Department of Pediatrics, Kurashiki Central Hospital, Kurashiki, 710-8602 Japan; 4 Department of Pediatrics, Yodogawa Christian Hospital, Osaka, 533-0024 Japan; 5 Division of Neonatology, Department of Pediatrics, Saitama Medical Center, Saitama Medical University, Kawagoe, 350-8850 Japan; 6 Department of Pediatrics, Osaka City University Graduate School of Medicine, Osaka, 545-8585 Japan; 7 Division of Neonatology, Center for Maternal-Neonatal Care, Nagoya University Hospital, Nagoya, 466-8560 Japan; 8 Graduate School of Medicine, Kyoto University, Kyoto, 606-8501 Japan; 9 Department of Pediatrics, The University of Tokyo, Tokyo, 113-8655 Japan; 10 Department of Neonatology, Osaka City General Hospital, Osaka, 534-0021 Japan; 11 Department of Pediatrics, Kyorin University, Mitaka, 181-8611 Japan

**Keywords:** Hypoxic-ischaemic encephalopathy, Translational research

## Abstract

Neonatal hypoxic-ischaemic encephalopathy (HIE) is a serious condition; many survivors develop neurological impairments, including cerebral palsy and intellectual disability. Preclinical studies show that the systemic administration of umbilical cord blood cells (UCBCs) is beneficial for neonatal HIE. We conducted a single-arm clinical study to examine the feasibility and safety of intravenous infusion of autologous UCBCs for newborns with HIE. When a neonate was born with severe asphyxia, the UCB was collected, volume-reduced, and divided into three doses. The processed UCB was infused at 12–24, 36–48, and 60–72 hours after the birth. The designed enrolment was six newborns. All six newborns received UCBC therapy strictly adhering to the study protocol together with therapeutic hypothermia. The physiological parameters and peripheral blood parameters did not change much between pre- and postinfusion. There were no serious adverse events that might be related to cell therapy. At 30 days of age, the six infants survived without circulatory or respiratory support. At 18 months of age, neurofunctional development was normal without any impairment in four infants and delayed with cerebral palsy in two infants. This pilot study shows that autologous UCBC therapy is feasible and safe.

## Introduction

Acute brain dysfunction may occur unexpectedly during and immediately after birth. Brain dysfunction noted immediately after birth, from any cause, is collectively termed neonatal encephalopathy^1^. The signs and symptoms of neonatal encephalopathy include altered levels of consciousness, weak muscle tone, impaired feeding, respiratory distress, and seizures. In many infants with neonatal encephalopathy, the pivotal pathophysiology is an insufficient supply of blood flow and oxygen to the brain in the ante-, intra-, or very immediate postpartum period. In these cases, neonatal encephalopathy is named hypoxic-ischaemic encephalopathy (HIE)^2^. Mild hypothermia is the only therapy proven effective for term newborns with HIE^3,4^. Even if infants with HIE receive therapeutic hypothermia, 40–44% die or survive with severe neurological impairments, including cerebral palsy and intellectual disability^3,4^. The therapeutic time window is within 6 hours after birth for hypothermia to be effective^3,5,6^. Hence, it is an urgent task to develop a novel therapy for HIE, especially a therapy with a long therapeutic time window^7^.

Cell-based therapy has attracted much attention because of not only its regenerative property but also its long therapeutic time window^8^. Preclinical studies with animal models with neonatal encephalopathy show that cell therapies initiated hours or days after the injury are beneficial. Although a variety of cell therapies have been reported as effective in preclinical studies, intravenous infusion of autologous (from the patient) umbilical cord blood cells (UCBCs) is currently regarded as the optimal cell therapy for newborns with HIE considering feasibility and safety^9,10^. When translating to the clinic, easy obtainability with no ethical problem in cell collection, no tumourigenicity, low immunogenicity, and easy preparation are critical.

Several preclinical studies on the systemic administration of UCBCs for neonatal HIE have been published thus far^11–25^. Most of these preclinical studies used the mononuclear cell (MNC) fraction of human UCBCs, and the cells were transfused either intraperitoneally or intravenously. Almost all studies demonstrated the beneficial effects of UCBC treatment, although publication bias cannot be ruled out. Our research group, named the Neonatal Encephalopathy Consortium Japan, conducted preclinical studies before translating UCBC therapy to the clinic. We demonstrated that the intravenous infusion of human CD34^+^ cells (haematopoietic stem/endothelial progenitor cells) at 48 hours after brain injury partially ameliorated the damage in mouse models of neonatal stroke^18^ and neonatal HIE^22^. Additionally, we have demonstrated that intraperitoneal infusion of the MNC fraction of human UCBCs at 6 hours after brain injury partially ameliorates the damage in a rat model of neonatal HIE^19^.

Regarding the intravenous infusion of autologous UCBCs for newborns with neonatal HIE, two clinical studies (NIH ClinicalTrials.gov website; NCT00593242, NCT01506258) apart from our study have been completed, and only one of these studies, which was conducted in the U.S., has been published^26^.

In this article, we present the protocol and results of our pilot study on the feasibility and safety of the intravenous administration of autologous UCBCs for newborns with HIE.

## Results

### Study outline

**Figure 1:**
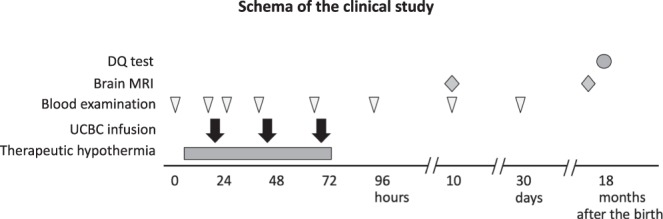
Schema of the clinical study: timing of the cell infusions and clinical examinations. DQ: developmental quotients, UCBCs: umbilical cord blood cells.

This clinical study strictly adhered to the study protocol as well as the standard operating procedure (SOP) for UCB processing, and there was no deviation from the protocol. The schema of this study is shown in Fig. 1. The review board of this multicentre clinical study approved proceeding to the third case after the interim assessment of the first two cases and then approved the adherence and safety and feasibility of this protocol after the final assessment of the six cases.

This clinical study started in 2015. The first case enrolment was in May 2015, and the final case enrolment occurred in September 2017. The reason why it took more than two years to enrol six cases was that we recruited only infants born *via* caesarean section in hospitals of our study group in order to secure the quality of UCB collection. As the majority of infants in Japan are born in private obstetrics clinics or small community hospitals, many HIE cases occur there and are then transferred to general hospitals with neonatal intensive care units (NICUs). All parents of eligible newborns, excluding one newborn, consented to their child’s enrolment in the study. We could not enrol that eligible case because the mother and grandmother had an intellectual disability and we could not get in contact with the father.

### Baseline characteristics of the cases

The characteristics of the six newborns are shown in Table 1. Four boys and two girls were enrolled. Three of these newborns were recruited at Kurashiki Central Hospital, and the remaining three were recruited at Yodogawa Christian Hospital, Saitama Medical Center, and Osaka City University Hospital. The causes of asphyxia were of foetal origin in four cases and maternal origin in two cases. Foetal causes included premature abruption of the placenta, prolapse of the cord, nonreassuring foetal distress due to an anomaly of rotation or difficulty of head ejection during birth. Maternal causes included cardiopulmonary arrest due probably to amniotic fluid embolism and loss of consciousness due to subarachnoid haemorrhage. The median pH of the UCB or first blood sample from the newborn was 7.16 (range, 6.96 to 7.23). The median base deficit of the UCB or first blood sample from the newborn was 8.4 (range, 5.1 to 20.0). Although the pH and base deficit of the UCB were not severely altered in Cases no. 1, no. 4, and no. 5, continued resuscitation for more than 10 minutes was needed; hence, these three cases met the inclusion criteria. Thorough examinations ruled out the possibility of diseases other than HIE, such as muscular diseases. The mothers of these two infants received general anaesthesia, but the clinical courses revealed that general anaesthesia was not the major cause of the continuous need for resuscitation. The Sarnat stage was II in five cases and III in one case. Therapeutic hypothermia was initiated by 4 hours after birth in all cases.

**Table 1:** Baseline clinical characteristics of six cases.

Sex	Case no. 1	Case no. 2	Case no. 3	Case no. 4	Case no. 5	Case no. 6
	Male	Male	Female	Male	Male	Female
Gestational age (weeks/days)	38w0d	40w0d	41w4d	39w5d	38w5d	39w5d
Birth weight (g)	2436	2507	3024	4086	2723	2727
Apgar score (1 min/5 min/10 min)	2/5/(15 min)8	0/0/1	2/2/3	5/6/9	2/7/7	1/3/5
Need for resuscitation>10 min	+	+	+	+	+	+
Couse of asphyxia	placental abruption	cord prolapse	mother’s CPA	non-reassuring fetal distress	non-reassuring fetal distress	mother’s LC
Cord blood analysis (artery)		*				
pH	7.225	6.963	7.132	7.230	7.195	7.065
base deficit (mmol/L)	7.4	20	9.3	5.1	10.3	6.0
PCO_2_ (mmHg)	48.2	69.5	59.6	53.7	45.1	84.5
lactate (mmol/L)	6.0	15	9.0	5.7	9.0	3.5
Sarnat staging	II	III	II	II	II	II
aEEG	moderately surpressed	severely surpressed	severely surpressed	moderately surpressed	moderately surpressed	moderately surpressed
Seizures	−	−	+	+	−	−

^*^Venous blood analysis, in which sample was obtained at 26 minutes after birth. CPA: cardiopulmonary arrest, LC: loss of consciousness.

### Cord blood data

In all cases, we attempted to collect UCB, and we succeeded in doing so. The volumes of collected UCB were 40–113 mL (Table 2). Total nucleated cell numbers after processing were 1.4–10.9 × 10^8^, which were equally divided into three doses and administered intravenously. The CD34^+^ cell doses administered were 0.3–9.7 × 10^6^. The retrieval rates of CD34^+^ cells after processing were more than 90% in three cases but less than 50% in two cases. In Case no. 1, the test sample of the processed cell solution was taken from the upper part of the solution after being maintained in a syringe for a period of time, which might have caused the retrieval rate to be low. In Case no. 2, the UCB volume before processing was 40 mL, which was the minimal volume for processing by an automated machine, and the low volume might have caused the retrieval rate to be low. The bacterial cultures of the processed UCB were negative in all six cases.

**Table 2:** Cord blood data.

		Case no. 1	Case no. 2	Case no. 3	Case no. 4	Case no. 5	Case no. 6
Cord blood collected	Collected volume (mL)	107	40	53	113	65	64.6
	Number of TNCs (× 10^6)	1,530	410	1,170	2,070	1,250	650
	Number of CD34^+^ cells (× 10^6)	5.5	0.9	3.1	10.5	6.7	1.8
Processed cord blood	Number of TNCs (× 10^6)	1,400	240	680	1,090	950	640
	Number of CD34^+^ cells (× 10^6)	1.8	0.3	2.4	9.7	6.6	2.2
	Lymphocytes (%)	31	49	46	35	8	41
	Neutrophiles (%)	61	40	47	49	51	46
	Monocytes (%)	8	7	5	10	8	11
Divided dose to infuse	Concentration of TNC (/μL)	77,800	13,300	37,800	60,600	52,800	35,600
	Concentration of CD34^+^ cell (/μL)	100	17	133	539	367	122

The data of three types of samples; 1) cord blood collected from a newborn, 2) the total dose of processed cord blood, and 3) each dose to be infused after dividing into three equal doses.

TNCs: total nucleated cells. Notably, the data in Case no. 1 may not be accurate, as the test sample of the processed cell solution was taken from the upper part of the solution after maintaining it in a syringe for a certain period of time.

### Cell transfusions and possible adverse events

**Figure 2:**
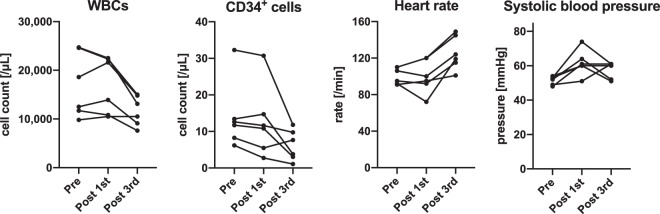
Temporal changes in leukocytes (WBCs) and CD34^+^ cells in peripheral blood samples, heart rate, and systolic blood pressure, immediately before the first cell infusion, at 2 hours after the first infusion, and at 24 hours after the third infusion.

The first infusion of processed UCB was performed between 16 hours and 22 hours after birth. There were no acute adverse events during the infusion or within the first few hours after the infusion. Table 3 presents the values in blood analyses and the physiological parameters at preinfusion (immediately before the first infusion), postfirst infusion (2 hours after the first infusion), and postthird infusion (24 hours after the third infusion). Figure 2 demonstrates the temporal changes in the major values and parameters. With respect to changes between preinfusion and postfirst infusion, there was no constant significant change in those values or parameters. Although C-reactive protein (CRP) levels were uniformly elevated after the first cell infusion, the elevation was minimal, namely, 0.3 mg/dL at maximum, and this temporal change was not statistically significant. The base deficit was exacerbated in four cases (2.9 mol/L increase at worst) but ameliorated in two cases. Systolic blood pressure was uniformly elevated after the first infusion; the median elevation was 10 mmHg, ranging from 2–21 mmHg. This temporal change, however, did not reach statistical significance. Neither the CD34^+^ cell counts nor the leukocyte counts in peripheral blood were elevated. With respect to changes between postfirst infusion and postthird infusion, there were significant changes in three of those values and parameters shown in Table 3; the base deficit decreased (improved), HCO_3_ increased, and the body temperature rose. The heart rate uniformly rose, but the temporal change did not reach statistical significance. None of these changes was considered to be caused by UCBC infusions but, rather, a result of the anticipated clinical course with medical care and cessation of therapeutic hypothermia.

**Table 3:** Changes in blood and physiological parameters between pre- and postfirst infusion and postthird infusion.

	Case no. 1			Case no. 2			Case no. 3			Case no. 4			Case no. 5			Case no. 6			temporal change	
	pre	post 1st	post 3rd	pre	post 1st	post 3rd	pre	post 1st	post 3rd	pre	post 1st	post 3rd	pre	post 1st	post 3rd	pre	post 1st	post 3rd	pre - post 1st	post 1st - 3rd
**Peripheral blood**																				
WBCs (/μL)	11,700	10,800	7,600	24,600	22,300	15,000	24,700	22,500	13,100	18,600	21,600	14,800	9,800	10,500	10,500	12,500	13,900	9,100	n.s.	n.s.
CD34^+^ cells (/μL)	11.7	10.8	3.04	13.4	14.7	3.78	8.26	5.48	7.65	32.3	30.7	11.8	12.6	11.6	9.75	6.19	2.74	1.09	n.s.	n.s.
Ht (%)	51.5	49.2	42.9	67.1	61.8	46.9	54.6	51.3	40.4	48.0	47.7	39.7	37.6	39.1	34.6	40.5	42.7	35.5	n.s.	n.s.
Plt (x10^3/μL)	171	185	115	266	260	191	233	229	214	220	218	245	187	208	183	207	240	168	n.s.	n.s.
CRP (mg/dL)	0.39	0.41	0.11	0.53	0.64	0.20	1.0	1.1	0.5	0.29	0.49		0.06	0.08	0.02	0.05	0.32	0.18	n.s.	n.s.
LDH (IU/L)	1,053	1,010	694	782	671	673	1,004	865	497	573	677	379	587	691	626	395	398	406	n.s.	n.s.
CK (IU/L)	2,059	2,133	441	1,139	1,090	1,257	402	298	84	1,275	1,731	143	247	247	103	221	221	97	n.s.	n.s.
K (mEq/L)	4.8	3.8	4.3	4.4	4.1	4.5	4.5	3.9	3.6	3.6	4.0	4.9	3.5	4.0	4.5	3.6	4.0	4.7	n.s.	n.s.
**Blood gas (artery)**																				
pH	7.439	7.407	7.331	7.313	7.338	7.421	7.294	7.340	7.302	7.370	7.393	7.352	7.372	7.400	7.419	7.334	7.307	7.450	n.s.	n.s.
PO_2_ (mmHg)	175	185	81.2	121	125	92.6	93.1	104	80.6	117	106	95.9	70.6	77.3	113	132	103	97	n.s.	n.s.
PCO_2_ (mmHg)	29	26.4	49.9	35.8	36.2	42.1	46.2	38.2	51	41.2	36.3	46.5	38.9	36.3	34.9	43.4	45.7	43	n.s.	n.s.
HCO_3_^-^ (mol/L)	19.6	16.6	26.3	17.6	18.9	26.8	21.7	20	24.5	23.8	22.1	25.8	22.6	22.5	22.6	22.1	21.2	29.2	n.s.	**
Base deficit (mol/L)	3.9	6.8	0.1	7.4	5.6	2.5	3.8	4.8	−1.1	1.5	2.5	0	2.5	2.0	−1.7	2.5	3.2	−5.5	n.s.	*
Body tempreature (°C)	33.5	33.5	36.2	33.5	33.5	36.8	34.0	34.0	36.8	33.6	33.5	37.2	33.5	33.5	36.8	33.4	33.6	37.2	n.s.	*
Heart rate (/min)	91	95	101	110	120	145	110	120	149	106	100	124	92	72	119	95	92	115	n.s.	n.s.
Diastolic blood pressure (mmHg)	38	45	37	40	38	38	36	39	32	37	53	35	31	35	36	35	41	44	n.s.	n.s.
Systolic blood pressure (mmHg)	52	64	52	49	51	61	54	60	51	53	74	61	48	61	61	53	60	60	n.s.	n.s.
Respirator mode	SIMV	SIMV	SIMV	SIMV	SIMV	SIMV	HFO	HFO	HFO	SIMV	SIMV	SIMV	SIMV	SIMV	SIMV	SIMV	SIMV	no respiratory support	n.s.	n.s.
FiO_2_ (%)	30	30	23	21	21	21	35	25	21	21	21	21	21	21	21	21	21	21	n.s.	n.s.
Rate (/min)	25	25	30	15	15	20				50	50	20	40	40	25	18	12		n.s.	n.s.
MAP (cmH_2_O)	6	6	6	5.5	5.5	6	12	12	11	6	6	6	6.3	6.2	6.2	6.1	6.1		n.s.	n.s.
PEEP (cmH_2_O)	5	5	5	5	5	5	SV15	SV 15	SV 16	5	5	5	5	5	5	5	5		n.s.	n.s.
Thompson score	15		12	12		15	9		9	12		12	15		15	9		0	n.s.	n.s.

pre: immediately before the first cell infusion, post 1st: at 2 hours after the first cell infusion, post 3rd: at 24 hours after the third cell infusion. SIMV: synchronized intermittent mandatory ventilation, HFO: high frequency oscillation, MAP: mean airway pressure, PEEP: positive end expiratory pressure, SV: stroke volume, n.s.: not significant, *significant change between preinfusion and postfirst infusion or postfirst infusion and postthird infusion. *P value < 0.05, **P value < 0.01.

Three infusions were successfully completed in each of six infants, and no adverse events were noted (Table 4). All infants treated with UCBCs became free from respiratory support, the use of vasopressors, or seizures by 10 days of age. The primary outcome measure was a combined rate of cases requiring cardiac support, respiratory support, or death at 30 days of age. Therefore, the primary outcome was zero out of six cases. Five infants were orally fed, and one infant was tube fed at 30 days of age. Other complications included seizures in two cases and hypotension, coagulopathy, weak cardiac muscle contraction, and hyperglycaemia in one case, all of which are common complications observed in infants with neonatal HIE treated with hypothermia and all of which responded to treatments. It is unlikely that those complications were the result of UCBC infusions.

**Table 4:** Outcomes and possible adverse events.

		Case no. 1	Case no. 2	Case no. 3	Case no. 4	Case no. 5	Case no. 6
24 h after the third infusion	Respiratory support	+	+	+	+	+	-
(91–95 hours of age)	Use of oxygen	+	−	−	−	−	−
	Use of vasopressor	no	no	no	+	+	+
	Thompson score	12	15	9	12	15	0
	Seizures	none	+	none	none	none	none
	Other adverse effect	none	none	none	none	none	none
10–13 days of age	Respiratory support	−	−	−	−	−	−
	Use of oxygen	+	+	−	−	−	−
	Use of vasopressor	no	no	no	no	no	no
	Head MRI	normal	T2 high intensity in bil. basal ganglia & thalamus	mild T1 high intensity in bil. basal ganglia & thalamus	normal	trivial IVH and subdural hemorrhage	normal
	Thompson score	0	11	3	0	0	0
	Seizures	none	none	none	none	none	none
	Other adverse effect	none	none	none	none	none	none
30 days of age	Respiratory support	−	−	−	−	−	−
	Use of oxygen	−	−	−	−	−	−
	Use of vasopressor	no	no	no	no	no	no
	Feeding	oral	tube feeding	oral	oral	oral	oral
	Seizures	no	no	no	no	no	no
	Anti-convulsant	no	no	no	no	no	no
	Other adverse effect	none	none	none	none	none	none
	Discharge	30 days of age	not yet	34 days of age	20 days of age	14 days of age	16 days of age
18 months of age	Medical care	none	tube feeding, use of oxygen	none	none	none	none
	Seizures	−	West syndrome	−	−	−	−
	Anti-convulsant	−	+	−	−	−	−
	CP	−	+	+	−	−	−
	MRI/CT	not examined	bil. lesions in basal ganglia & thalamus, brain atrophy	bil. lesions in basal ganglia & thalamus	normal	normal	normal
	DQ score	88	no head control, no pursuit objects	55	110	109	108
	Postural-Motor	102		47	109	95	131
	Cognitive-Adaptive	85		58	107	109	96
	Language-Social	89		48	117	116	122

DQ scores were evaluated by the Kyoto Scale of Psychological Development. bil.: bilateral, IVH: intraventricular haemorrhage, CP: cerebral palsy.

### Neurological outcomes

The secondary outcome measures were neuroimaging at 12–18 months of age and neurodevelopmental level at 18 months of age. Brain MRI showed abnormalities in the bilateral basal ganglia and thalamus in two infants and no abnormalities in four infants (Table 4). Brain MRI at 10–13 days after birth showed the same abnormalities in the bilateral basal ganglia and thalamus in two infants, trivial intraventricular and subdural haemorrhage in one infant, and no abnormality in the remaining three infants (Table 4).

All six infants were examined by the Kyoto Scale of Psychological Development at 18 months of age^27^, which is the most widely used test for neurological development in Japan, and no infant was examined via Bayley III. Four infants were in good physical condition without any sequelae at 18 months of age (Table 4). The developmental quotients (DQs) for these infants were higher than 85 (range, 88–110). Two other infants had neurofunctional impairments and developmental delays. Case no. 2 had spastic cerebral palsy and profound developmental delay, that is, he could not control his head or pursue objects. This infant could not be fed orally and was therefore fed via a catheter set by percutaneous endoscopic gastrostomy. He exhibited West syndrome, and his respiratory condition was not stable, necessitating the use of oxygen and respiratory support with nasal bilevel positive airway pressure during night sleep. The general condition of Case no. 3 was good, although this infant had mild spastic cerebral palsy. She could sit and stand up alone but could not walk yet. Her DQ was 55.

## Discussion

The aim of this clinical study was to examine the feasibility and safety of autologous UCBC infusion in newborns with HIE. Our treatment protocol prioritized the feasibility foremost. The cell doses administered were not adjusted and inherently determined by the volumes of collected UCB and the numbers of cells in it, as autologous UCB was used. The timings of cell infusion were extrapolated from animal studies considering the safety of noncryopreserved UCB. Some of the data of this clinical study have already been published, i.e., the brief data on early time points from five of the six cases^28^. In this article, we have presented more detailed data up to 18 months of age from all six cases.

We assumed that the following two issues might be crucial in this study: (1) collecting a sufficient volume of UCB in clinical emergencies, i.e., birth of critically ill newborns with asphyxia, and (2) the sterility of the UCB. In all cases, we tried to collect UCB, and we successfully collected an adequate volume of it with no bacterial contamination. There were no problems in volume-reducing processing with the automated centrifugation machine or preserving the processed UCB until use. The retrieval rates of CD34^+^ cells after processing were less than 50% in the first two cases. The actual retrieval rate in Case no. 1 was not that low, as the test sample of the processed UCB was drawn out of the upper part of the UCB kept in a syringe for a certain period, i.e., we assumed that the upper part of the UCB might have become like a supernatant with fewer cells. We shared this information among our study group members. Subsequently, test samples were drawn after mixing the UCB in a syringe. The low retrieval rate of CD34^+^ cells in Case no. 2 was probably due to the fact that the UCB volume before processing was the lowest possible volume for automated processing, which might have caused many CD34^+^ cells to be removed from the final UCB product together with red blood cells (RBCs). A study showed that small volumes of bone marrow resulted in lower leukocyte recovery by the automated machine Sepax^® ^^29^. In the other four cases, the retrieval rates were sufficiently high, 77% in one case and more than 90% in the other three cases.

The main safety issue in this study was whether the physiological condition, especially circulatory and respiratory conditions, in severely ill infants would be stable during and after cell infusions. All six infants were sick in the perinatal period and received 72 hours of whole-body cooling initiated by 4 hours after birth; therefore, these infants were vulnerable to factors that might influence cardiovascular and respiratory status. We closely monitored the cardiovascular and respiratory status during the first several days of cell therapy. We did not find changes that might be caused by cell infusions. Additionally, we repeatedly analysed peripheral blood during the first several days of cell therapy. Even though the infants received concentrated UBC, the blood cell counts were not altered during the cell therapy period. CD34^+^ cell counts did not increase following UCB infusion, which was not unexpected as physiologically CD34^+^ cell counts rapidly decrease over the first few days of life^30,31^. Returning 0.3–9.7 × 10^6^ CD34^+^ cells did not prevent a decrease in the cell concentration in the peripheral blood.

As the cells were autologous UCBCs with minimal processing, the tumourigenicity of the infused cells was not an issue. Therefore, the primary outcome measure of this study was set as a combined rate of circulatory stability, respiratory stability, and death at 30 days of age. All six participants of this study survived up to the end of the observation period, i.e., 18 months of age. There was not a single case that required circulatory support, such as the use of vasopressors, or respiratory support, such as the use of oxygen at 30 days of age. However, one infant later required the use of oxygen and respiratory support during night sleep.

Recently, the Baby Cooling Registry of Japan Collaboration Team reported data collected from 485 HIE infants who received therapeutic hypothermia from 2012 to 2014^32^. The basic characteristics of each of the study participants are similar, although the severity of HIE was slightly milder in the present study (Table 5). The rates of adverse events in the present study were similar to or somewhat milder than those in the report. The report did not include neurological outcomes, such as the mean DQ and rate of cerebral palsy.

**Table 5:** Comparison of the present study and a recent cohort study.

	Present study	Reference*
**Apgar score**		
1 min	2 (0–5)	1 {1–3}
5 min	4 (0–7)	4 {2–5}
10 min	6 (1–9)	5 {3–7}
**Cord or first glood gas**		
pH	7.16 (6.96–7.23)	6.94 ± 0.21
base deficit (mmol/L)	8.4 (5.1–20.0)	14.6 ± 10.5
**Sarnat staging**		
stage I	0	11.7
stage II	83	61.1
stage III	17	27.2
Hypotension	17	34.8
Seizures	33	24.7
Coagulation disorders	17	13.2
Arrythmia	0	1.4
Weak cardiac muscle contraction	17	n/a
Hyperglycemia	17	n/a
Hypoglycemia	0	1
Septicemia	0	0.8
Subcutaneous fat necrosis	0	0.4
Dependence on tube feeding	17	18.6
Dependence on respiratory support	0	9.7
Death before discharge	0	2.7

Reference; Tsuda *et al*., *Sci Rep* 2017.

The Apgar scores are presented as the median and range in the present study and as the median and interquartile range in the reference study. The data from blood examinations are presented as the median and range in the present study and as the mean ± standard deviation in the reference study. All other data are presented as the ratio (%) of cases among study participants. n/a: data not available.

To the best of our knowledge, three clinical studies, including ours, on the intravenous infusion of autologous UCBC therapy for neonatal encephalopathy have been completed according to the NIH website, ClinicalTrials.gov: ClinicalTrials.gov Identifier NCT02256618 (ours), NCT (Singapore) and NCT00593242 (USA). Only one (NCT00593242) of these studies has been published^26^. This study was a single arm study with 23 infants treated with the intravenous infusion of volume-reduced noncryopreserved UCBCs. No severe adverse events that might be associated with the cell infusions were observed. The outcome was similar to ours. The feasibility and safety of the intravenous infusion of cryopreserved autologous UCB have been reported in a series of studies in children with cerebral palsy^33,34^.

The present study was a small single arm study. Therefore, it is not possible to evaluate the beneficial effect of UCBC therapy. Four out of six infants who received therapy survived without any noticeable sequelae and with a DQ higher than 85, while the remaining two infants developed cerebral palsy, and one of the two infants had a profound intellectual disability with West syndrome. This infant with the most severe sequelae could have been worse, as his Apgar score was 1 at 10 minutes after birth and the Sarnat stage was grade III.

This treatment protocol may not be the most effective one. Further preclinical studies are needed to optimize the treatment protocol. To the best of our knowledge, no preclinical study has thoroughly examined the optimal timing of UCBC infusion in models with neonatal brain injuries. In the literature, almost all preclinical studies on neonatal brain injuries examined the effects of UCBC administration at only one timepoint. Many such studies reported beneficial effects of UCBC administration at 24 or 48 hours after brain injury^11,12,15,17,18,22,24,25,35^. Preclinical studies on other types of cells and/or in other models have demonstrated that early administration, i.e., a few hours after brain injury is not as beneficial as later administration, such as 24–72 hours after brain injury^36^. Similarly, much later administration, such as later than 10 days after brain injury, is not beneficial either^36,37^. Moreover, there is no preclinical evidence to guide the combined hypothermia plus UCBC treatment; all preclinical studies with UCBCs examined the effects of cell treatment alone. Preclinical studies on mesenchymal stem cells (MSCs) reported contradicting interactions with hypothermia. Herz *et al*. reported detrimental interactions between hypothermia and delayed murine bone marrow-derived MSC therapy^38^, while Park, Ahn, *et al*. reported benefits when human UCB-derived MSCs were administered pre-^39^ and post-hypothermia^40^.

The feasibility and safety of the intravenous transfusion of autologous UCBCs for newborns with HIE along with therapeutic hypothermia was shown in this pilot study. The next step is to examine the safety and efficacy of this therapy in a randomized control study with a larger number of patients.

## Methods

We designed a protocol for our clinical study based on our preclinical studies and taking the feasibility of the cell therapy into consideration. Our protocol was mostly adjusted to that of the published clinical study in the U.S.^26^, as we may be able to collaborate in conducting a large-scale international phase 3 trial in the future. This clinical study was carried out in accordance with relevant guidelines and regulations, including the Declaration of Helsinki and the Act on Securement of Safety of Regenerative Medicine, Japan. Our protocol and other related documents, including an informed consent form for parents, were approved by the Ethics Committees of Osaka City University Graduate School of Medicine and institutional review boards (IRBs) of six other participating institutes. Additionally, our protocol and related documents were approved by the Certified Committee for Regenerative Medicine set by the Ministry of Health, Labor and Welfare of Japan. Our study was registered at the NIH ClinicalTrials.gov website (ClinicalTrials.gov Identifier: NCT02256618; date of registration, 3rd of October, 2014) and the UMIN (University Hospital Medical Information Network Japan) website.

This study was a multicentre pilot study to evaluate the feasibility and safety of the intravenous infusion of autologous UCBCs in term newborns with HIE. The study was an open-label, single-group assignment, and the number of planned enrolments was six.

The participants in this study were recruited from seven NICUs in Japan: Kurashiki Central Hospital, Yodogawa Christian Hospital, Saitama Medical Center, Osaka City University Hospital, Osaka City General Hospital, Nagoya University Hospital, and The University of Tokyo Hospital. All pregnant women admitted to the hospitals for delivery received explanations of a possible case of UCB collection for this clinical study in advance, and verbal consent was obtained. If their babies were born by caesarean section due to foetal distress or other medical problems and if the infants had severe asphyxia, then their UCB were collected with special care to avoid contamination. Immediately after the placenta was delivered, the umbilical cord was disinfected with iodine solution. A needle connected to a tube and to a collection bag was inserted into the umbilical vain so that the UCB spontaneously drained into the collection bag containing an anticoagulant, namely, citrate-phosphate-dextrose-adenine (CPDA), inside. Then, the UCB was stored at 4 °C. If a neonate presented with signs and symptoms of moderate to severe encephalopathy and met the criteria for therapeutic hypothermia, then whole-body cooling (33.5 °C) was initiated by 4 hours after the birth, and the neonate was considered for enrolment into this clinical study. The inclusion and exclusion criteria for this study were consistent with those of therapeutic hypothermia for term neonates with HIE (the International Liaison Committee on Resuscitation (ILCPOR) Consensus on Science and Treatment Recommendation (CoSTR) 2010 guideline)^41^. Those criteria are shown below (of note, these criteria are listed on the NIH ClinicalTrial.gov website and UMIN website^42^):

Inclusion criteria:

Infants were eligible if they met all the following inclusion criteria, except 4):

1. ≥36 weeks gestation
2. Either a 10-minute Apgar score ≤5, continued need for resuscitation for at least 10 minutes, or severe acidosis, defined as pH < 7.0 or base deficit ≥ 16 mmol/L in a sample of UCB or any blood during the first hour after birth
3. Moderate to severe encephalopathy (Sarnat II to III)
4. A moderately or severely abnormal background amplitude-integrated EEG (aEEG) voltage, or seizures identified by aEEG, if monitored
5. Up to 24 hours of age
6. Autologous umbilical cord blood available to infuse within 3 days after birth
7. A person with parental authority must have consented to the study.

The exclusion criteria were as follows:

1. Known major congenital anomalies, such as chromosomal anomalies and heart diseases
2. Major intracranial haemorrhage identified by brain ultrasonography or computed tomography
3. Severe growth restriction, with a birthweight of less than 1800 g
4. Severe infectious diseases, such as sepsis
5. Hyperkalaemia
6. Infants born at hospitals or clinics other than the study sites
7. Vaginal delivery
8. Volume of collected cord blood <40 mL
9. Infants judged critically ill and unlikely to benefit from neonatal intensive care by the attending neonatologist

Soon after a newborn was confirmed to meet the criteria by the initial assessment, the physicians explained the clinical study to the parents. When the parents agreed to participate in the study, written parental consent was obtained twice: after the first explanation of the study, which was a few hours after birth, and before the first UCBC infusion, which was approximately 20 hours after birth.

RBCs in the UCB were removed, and the volume was reduced to 20 mL by centrifugation using an automated machine, Sepax^®^ (Biosafe, Inc., Switzerland). UCB and hydroxyl ethyl starch (HES) (NIPRO Co., Ltd., Osaka, Japan) to remove RBCs were introduced into a rotating syringe^43^. After centrifugation, the volume-reduced UCB was introduced into a set of three collection bags. All the processes were automatically managed by the manufacturer’s software and performed in a closed disposable kit provided by the manufacturers (Biosafe, Inc., and TERUMO Co., Ltd., Tokyo, Japan). The minimum amount of collected UCB for the inclusion criteria of this trial was 40 mL, as automated UCB processing may not be reliable if the processing volume is less than 40 mL. Processed UCB contains all types of nucleated cells, including a variety of stem cells, e.g., CD34^+^ haematopoietic stem/endothelial progenitor cells. Of note, the number of RBCs was significantly reduced but not completely eliminated from the final UCB solution to be infused. The processed UCB was stored at 4 °C until use. The cell dose was not adjusted in each case. Previously, we evaluated the quality of the processed UCB using UCB collected from volunteers and confirmed the sterility, low haemolysis, and high cell viability, e.g., the viability of CD34^+^ cells was more than 90% at up to 96 hours after cell processing and storage at 4 °C of the samples^28^. Processed UCB was divided into three doses and intravenously administered at 12–24, 36–48, and 60–72 hours after the birth (Fig. 1). Before cell infusion, hydrocortisone (1 mg/kg body weight) was intravenously administered to reduce the possible risk of allergic reactions. As CPDA and HES were added to UCB, the risk of allergic reaction was not completely negligible even though the UCB was autologous. Each cell infusion was 6 mL in volume and 1 hour in duration using an infusion pump. No vehicle was used for delivery of the processed UCB. During the infusion, the processed UCB in a syringe was sometimes stirred by turning and rotating the syringe to avoid sedimentation of cells in the UCB. The condition of the infant, especially their circulatory and respiratory status, was closely monitored during and after the cell infusions. We set the primary outcome measure as the rate of adverse events, namely, the combined rate of three adverse events: death, continuous need of respiratory support, and continuous use of vasopressors at 30 days of age. The secondary outcome measure was efficacy, namely, neuroimaging at 12–18 months of age and neurodevelopmental level evaluated with the Bayley III or Kyoto Scale of Psychological Development at 18 months of age^27^.

Statistical analysis was performed for the values in blood analyses and physiological parameters shown in Table 3. Temporal changes in each value or parameter among three time points, i.e., immediately before the first UCBC infusion, 2 hours after the first UCBC infusion, and 24 hours after the third UCBC infusion were analysed by Kruskal-Wallis test followed by Dunn’s post hoc test. The Thompson score was analysed by the Mann-Whitney *U*-test, as the score was evaluated at two time points, i.e., immediately before the first infusion and 24 hours after the third infusion. A *P* value < 0.05 was considered statistically significant.

## Data Availability

The datasets generated and analyzed during the current study are available from the corresponding authors on reasonable request.
